# Anticancer Potential of *Sutherlandia frutescens* and *Xysmalobium undulatum* in LS180 Colorectal Cancer Mini-Tumors

**DOI:** 10.3390/molecules26030605

**Published:** 2021-01-25

**Authors:** Chrisna Gouws, Tanya Smit, Clarissa Willers, Hanna Svitina, Carlemi Calitz, Krzysztof Wrzesinski

**Affiliations:** 1 Pharmacen™, Centre of Excellence for Pharmaceutical Sciences, North-West University, Private Bag X6001, Potchefstroom 2520, South Africa; tanyasmit.smit@gmail.com (T.S.); clarissa.willers@nwu.ac.za (C.W.); 30884365@nwu.ac.za (H.S.); kwr@celvivo.com (K.W.); 2 Department of Medical Cell Biology, Uppsala University, Box 571, Husargatan 3, 75431 Uppsala, Sweden; carlemi.calitz@mcb.uu.se; 3 CelVivo ApS, 5491 Blommenslyst, Denmark

**Keywords:** anticancer, colorectal cancer, functional spheroids, phytomedicine, sodium alginate, *Sutherlandia frutescens*, three-dimensional cell culture, *Xysmalobium undulatum*

## Abstract

Colorectal cancer remains to be one of the leading causes of death worldwide, with millions of patients diagnosed each year. Although chemotherapeutic drugs are routinely used to treat cancer, these treatments have severe side effects. As a result, the use of herbal medicines has gained increasing popularity as a treatment for cancer. In this study, two South African medicinal plants widely used to treat various diseases, *Sutherlandia frutescens* and *Xysmalobium undulatum,* were evaluated for potential activity against colorectal cancer. This potential activity for the treatment of colorectal cancer was assessed relative to the known chemotherapeutic drug, paclitaxel. The cytotoxic activity was considered in an advanced three-dimensional (3D) sodium alginate encapsulated LS180 colorectal cancer functional spheroid model, cultured in clinostat-based rotating bioreactors. The LS180 cell mini-tumors were treated for 96 h with two concentrations of each of the crude aqueous extracts or paclitaxel. *S. frutescens* extract markedly decreased the soluble protein content, while decreasing ATP and AK per protein content to below detectable limits after only 24 h exposure. *X. undulatum* extract also decreased the soluble protein content, cell viability, and glucose consumption. The results suggested that the two phytomedicines have potential to become a source of new treatments against colorectal cancer.

## 1. Introduction

Cancer remains a global burden despite the medical advances made every day, with an estimated 9.6 million deaths worldwide in 2018. Colorectal cancer specifically accounts for 1.8 million cases annually, with approximately 70% of deaths occurring in low- and middle-income countries [1]. Colon cancer is often only detected when symptoms appear during advanced stages, and standard treatment includes surgery, chemotherapy, radiation therapy, immunotherapy, and nutritional support therapy [2]. Although surgical resection is the primary treatment option for colorectal cancer, more than half of the patients present with metastasis. Improving survival rates of patients with advanced colorectal cancer while providing symptomatic treatment are attempted with chemotherapy [3]. However, the risk for chemotherapeutic toxicity increases in older patients, with side effects of varying severity [4,5].

Patients suffering from this disease are usually desperate for a cure, or simply to reduce the severe side effects of conventional treatment. To this end, they frequently turn to alternative treatments, which typically include plant-based medicines or phytomedicines [6]. Although these treatments may be curative or beneficial, they may also have adverse effects and remain unproven. However, plants have been the source of new, clinically significant anticancer compounds, and 60% of anticancer agents currently used were derived from natural sources [7].

Two South African medicinal plants were identified as potential sources to improve treatment of colorectal cancer. *Sutherlandia frutescens*, commonly known as cancer bush, have been claimed to have anticancer properties by traditional healers, and these claims have been partially validated [8,9]. Several studies have also confirmed that no physiological toxicity is associated with the use of *S. frutescens* [10,11]. *Xysmalobium undulatum*, also known as Uzara, is one of the most widely used phytomedicines in South Africa [12], and is known to contain plant cardenolides which have emerged as promising new cancer treating agents [13]. Although a few studies have shown some potential anticancer effects of *X. undulatum in vitro*, it is not clear if the effects were cancer-specific or if the extracts are cytotoxic in general at high concentrations [14,15,16]. These two plants were selected as one has been studied extensively and the other only very limited.

The standard drug development pipeline must still be followed when studying phytomedicines. However, only an estimated 3.4% of new cancer drug candidates are successful in clinical trials [17]. This is probably due to the lack of physiological relevance of the current models used in this pipeline. The use of animal models in research is also expensive and ethically challenging [18]. Clearly, better in vitro models with high throughput capacity is crucial for cancer treatment screening [19]. It has been suggested that three-dimensional (3D) cell culture models are ideal to bridge the gap between in vitro and in vivo preclinical studies, due to their better correlation to human physiology and ability to mimic the microenvironment of cells and organs in vivo [20,21].

We recently developed and validated a sodium alginate encapsulated 3D colorectal cancer cell culture model, by culturing LS180 cells using the dynamic clinostat bioreactor technique [22]. We demonstrated the reactivity of the model to anticancer treatments, and its suitability as an in vitro screening tool to evaluate potential treatments for colorectal cancer. In the previous publication, we demonstrated that we bioengineered the cell constructs in such a way that the in vitro model presents in vivo-like behavior, therefore referred to as functional spheroids or mini-tumors [22].

The aim of this study was to use this new LS180 functional spheroid model to evaluate the potential of crude aqueous extracts of *S. frutescens* and *X. undulatum* as anticancer treatments for colorectal cancer. This was attempted by establishing the 50% inhibitory concentrations (IC_50_) of the selected phytomedicines in LS180 cells cultured as two-dimensional (2D) monolayers, using the 3-(4,5-dimethylthiazol-2-yl)-2,5-diphenyltetrazolium bromide (MTT) cell viability assay. Then, 50 12-day-old sodium alginate encapsulated spheroids per biological replicate were treated for 96 h with these IC_50_ concentrations and either half that dose ([IC_50_]/2 for *S. frutescens*), or two times the IC_50_ concentration (2[IC_50_] for *X. undulatum*). This was dependent on the maximal solubility achievable for each extract. During the treatment, various parameters were measured as indicators of cell viability. These parameters included intracellular adenosine triphosphate (ATP) content, extracellular adenylate kinase (AK) release, glucose consumption, and cell growth in terms of soluble protein content were also measured. Relative gene expression of some cytochrome P450 (CYP450) metabolic enzymes was measured using a real-time reverse transcription polymerase chain reaction-based (qRT-PCR) assay. These enzymes can directly affect bioavailability and drug resistance.

We established that the *S. frutescens* and *X. undulatum* crude aqueous extracts both showed notable cytotoxic effects and potential anticancer activity relative to the known chemotherapeutic drug paclitaxel, with decreased growth, cell viability, and glucose consumption following treatment.

## 2. Results

### 2.1. Preparation and Characterization of the Plant Material Aqueous Extracts

*X. undulatum* (XU174) and *S. frutescens* (SFFW) plant material (dried and milled) were purchased, and crude aqueous extracts were prepared. The plant extracts were chemically characterized with ultra-performance liquid chromatography-mass spectrometer (UPLC-MS) analyses.

**Figure 1:**
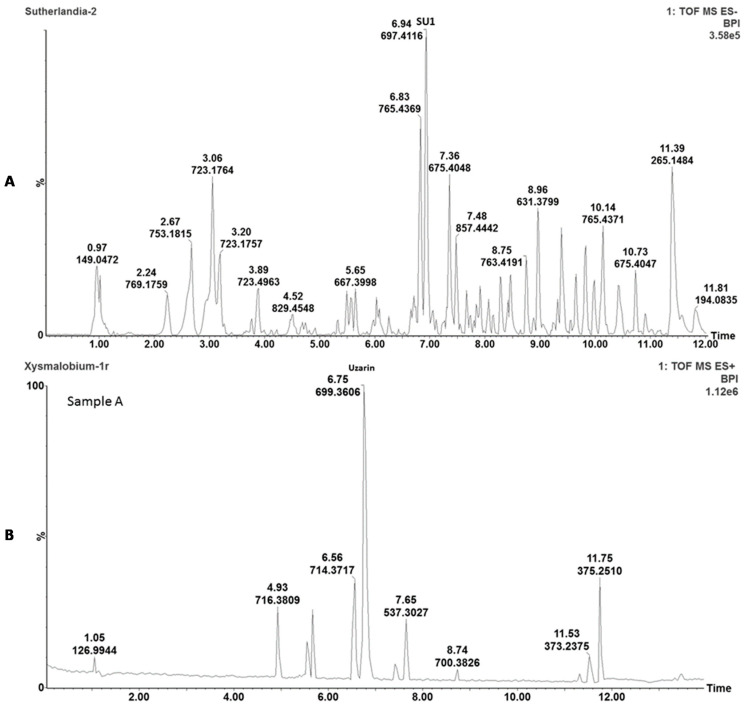
The LC-MS chromatogram of the crude aqueous *Sutherlandia frutescens* extract (**A**), and the UPLC chromatogram of the crude aqueous *Xysmalobium undulatum* extract (**B**).

After UPLC-MS analysis of *S. frutescens* (see Figure 1A), the cycloartane-like triterpene glycoside (SU1) marker molecule was identified, and quantified as 10.1 µg.mg^−1^ (*n* = 2) [15]. The UPLC-MS chromatogram of *X. undulatum* aqueous extract (see Figure 1B) confirmed the presence of the main marker molecule, uzarin, in the extract.

SU1, also known as sutherlandioside B, is stated to be the main compound present in commercial *S. frutescens* material, although other compounds present include pinitol, flavonoids, triterpenoids, polysaccharides, canavanine and amino acids such as the neurotransmittor Gamma aminobutyric acid (GABA) [8].

The major compounds present in *X. undulatum* roots are uzarin and xysmalorin, with other compounds including allouzarin, alloxysmalorin, and uzarigenin [8].

### 2.2. Pre-Screening of Anticancer Activity

The MTT assay was applied to evaluate the anticancer activity of the plant extracts and the chemotherapeutic drug, paclitaxel, in LS180 cells cultured as a traditional monolayer (see Figures S1–S3). Subsequently, the Probit analysis was applied to estimate the IC_50_ of each treatment, relative to an untreated control [23]. The determined IC_50_ values and their 95% confidence intervals are presented in Table 1.

The LS180 colorectal cell line demonstrated reduced cell viability relative to the untreated control following treatment for 96 h with paclitaxel, *S. frutescens* and *X. undulatum*. The IC_50_ concentration for paclitaxel was calculated as 8.10 × 10^−5^ mg·mL^−1^ for the LS180 cell line [22], while it was 2.63 mg·mL^−1^ for *S. frutescens* and 9.80 × 10^−2^ mg·mL^−1^ for *X. undulatum*.

These IC_50_ values were then used to screen the anticancer potential of the plant extracts in the LS180 sodium alginate encapsulated functional spheroid model, relative to paclitaxel.

### 2.3. Sutherlandia frutescens Anticancer Activity Screening in the LS180 Sodium Alginate Encapsulated Spheroid Model

We investigated the effect of 96 h exposure to [IC_50_] and [IC_50_]/2 *S. frutescens*, replaced every 24 h, on the growth and viability of 12-day-old LS180 spheroids. The crude aqueous *S. frutescens* extract was not soluble at the calculated double [IC_50_] concentration, and was therefore excluded from the study. It was then substituted with [IC_50_]/2 in the second biological repeat. The evaluations included the soluble protein content, intracellular ATP content, extracellular AK release, and glucose consumption. These parameters were then compared with the same parameters measured in LS180 spheroids treated with [IC_50_] paclitaxel, the results of which were previously published in Smit et al. [22].

#### 2.3.1. Soluble Protein Content

**Figure 2:**
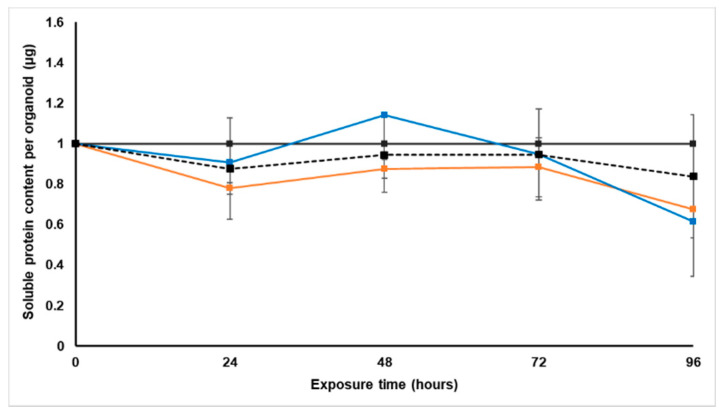
Normalized soluble protein content per spheroid (µg) of the sodium alginate encapsulated LS180 mini-tumor model, following 96 h exposure to *Sutherlandia frutescens* aqueous extract or paclitaxel. The solid black line represents the untreated control, the black dashed line represents paclitaxel [IC_50_], the orange line represents *S. frutescens* [IC_50_], and the blue line *S. frutescens* [IC_50_]/2. All data were normalized to the untreated control group (error bars = standard deviation, *n* = 3 for *S. frutescens* [IC_50_]/2 and *n =* 6 for *S. frutescens* [IC_50_] and paclitaxel [IC_50_]).

The soluble protein content per spheroid was measured every 24 h using the Bradford assay, and all data were normalized to the untreated control group (Figure 2). The protein content is an indicator of cell growth, but was also used to adjust the daily doses per protein content.

Although the soluble protein content per spheroid of the paclitaxel-treated group was lower than that of the untreated spheroids, this was not significant [22]. Treatment with *S. frutescens* for 24 h slightly reduced the soluble protein content in the LS180 spheroids in a concentration-dependent manner, followed by an increase in protein content. This increase was, however, less pronounced for the group treated with the higher dose of *S. frutescens* ([IC_50_]). Over the next 48 h the soluble protein content of both *S. frutescens*-treated groups decreased notably to almost 0.4 µg per spheroid less than the untreated control group. This decrease was also more pronounced than that of the paclitaxel-treated group.

The total soluble protein content per bioreactor was determined before each new dosing to ensure constant exposure of all spheroids to the same amount of treatment, irrespective of spheroid removal or growth. The dosages per protein was established as 0.60 × 10^−2^ µg·mL^−1^·µg^−1^ protein for [IC_50_] paclitaxel, 20.10 × 10^−2^ mg·mL^−1^·µg^−1^ protein for [IC_50_]/2 *S. frutescens* and 40.3 × 10^−2^ mg·mL^−1^·µg^−1^ protein for [IC_50_] *S. frutescens*. By multiplying these values by the amount of soluble protein per spheroid based on the number of spheroids left in the bioreactor for that specific day, the dosage per bioreactor per day could be determined. This enables better comparison to blood plasma levels of in vivo studies, since the IC_50_ (mg·mL^−1^) is converted to LD_50_ (mg compound per mg cellular protein) [24].

#### 2.3.2. Intracellular Adenosine Triphosphate Content

The intracellular ATP levels were determined every 24 h during treatment with paclitaxel and *S. frutescens*, with the CellTiter-Glo^®^ luminescence assay. These levels were then expressed per µg protein, and normalized relative to the untreated control group.

**Figure 3:**
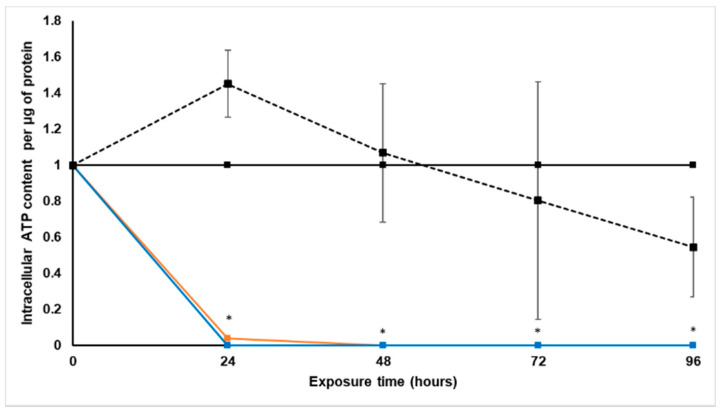
Normalized intracellular adenosine triphosphate content per soluble protein (µM·µg^−1^) following exposure of the sodium alginate encapsulated LS180 mini-tumor model to *Sutherlandia frutescens* aqueous extract and paclitaxel. The solid black line represents the untreated control, the black dashed line represents paclitaxel [IC_50_], the orange line represents *S. frutescens* [IC_50_] and the blue line *S. frutescens* [IC_50_]/2. All data were normalized to the untreated control group (error bars = standard deviation; *n* = 3 for *S. frutescens* [IC_50_]/2; *n* = 6 for *S. frutescens* [IC_50_] and paclitaxel [IC_50_]; * = statistically significant, *p* < 0.05 (one-way ANOVA followed by the Dunnett post-hoc test).

As seen in Figure 3, the spheroids treated with [IC_50_] paclitaxel for 24 h had a slight increase in intracellular ATP relative to the untreated control spheroids. Beyond 24 h treatment, the paclitaxel group showed a steady decline in intracellular ATP until being markedly lower than the untreated control at 96 h. This decrease suggests that the treatment with paclitaxel affected the viability and energy status of the LS180 cells negatively [22].

After 24 h of treatment with both *S. frutescens* concentrations, intracellular ATP content per protein was decreased significantly, since no ATP could be detected in the spheroids. These undetectable levels of ATP remained as such for the duration of the experiment for both treatment groups, relative to the untreated control. The intracellular ATP content per protein also decreased much faster, and was also lower than that of the group treated with paclitaxel.

This significant depletion of intracellular ATP is indicative of a loss of cell viability. We subsequently incubated the remaining LS180 spheroids for a further 96 h with no treatment to measure possible recovery, but the intracellular ATP remained below detectable limits (results not shown).

#### 2.3.3. Extracellular Adenylate Kinase Content

**Figure 4:**
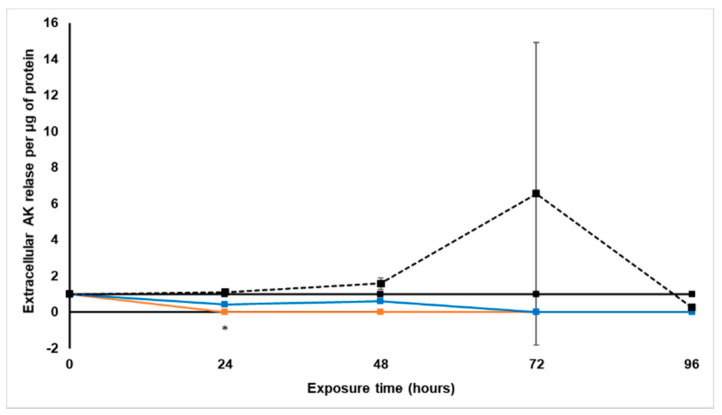
Normalized extracellular adenylate kinase release per microgram protein following exposure of the sodium alginate encapsulated LS180 mini-tumor model to *Sutherlandia frutescens* aqueous extract and paclitaxel. The solid black line represents the untreated control, the black dashed line represents paclitaxel [IC_50_], the orange line represents *S. frutescens* [IC_50_] and the blue line *S. frutescens* [IC_50_]/2. All data were normalized to the untreated control group (error bars = standard deviation; *p* < 3 for *S. frutescens* [IC_50_]/2; *n* = 6 for *S. frutescens* [IC_50_] and paclitaxel [IC_50_]; * = statistically significant, *p* < 0.05 (one-way ANOVA followed by the Dunnett post-hoc test).

The extracellular AK levels per protein content were determined with the ToxiLight^®^ luminescence BioAssay every 24 h during treatment with *S. frutescens* [IC_50_]/2, *S. frutescens* [IC_50_] and paclitaxel [IC_50_] for 96 h. These AK levels were then expressed relative to the untreated control, as presented in Figure 4.

Although no considerable changes in the extracellular AK levels occurred relative to the untreated control during the first 48 h of treatment with paclitaxel, there was a rapid increase of extracellular AK after 72 h treatment, followed by a decrease in the following 24 h to levels slightly lower than the untreated control group. This suggests that all cells were already dead by 96 h, and could no longer release AK [22].

Exposure to the *S. frutescens* aqueous extract for 24 h significantly decreased extracellular AK release below detectable limits for the *S. frutescens* [IC_50_] group (*p* < 0.05), and these levels stayed as such for the duration of the experiment. The *S. frutescens* [IC_50_]/2 group also showed a decrease in AK per µg protein at the 24 h time point, but this decrease was not significant. Although the AK release increased very slightly at 48 h, it then also decreased below detectable limits from 72 h onwards relative to the untreated control group.

The results suggest that both concentrations of *S. frutescens* had a faster and much more pronounced effect on AK release than paclitaxel, and if AK was released by the *S. frutescens* [IC_50_] group, this would have had to take place within the first 24 h of treatment.

#### 2.3.4. Glucose Consumption

**Figure 5:**
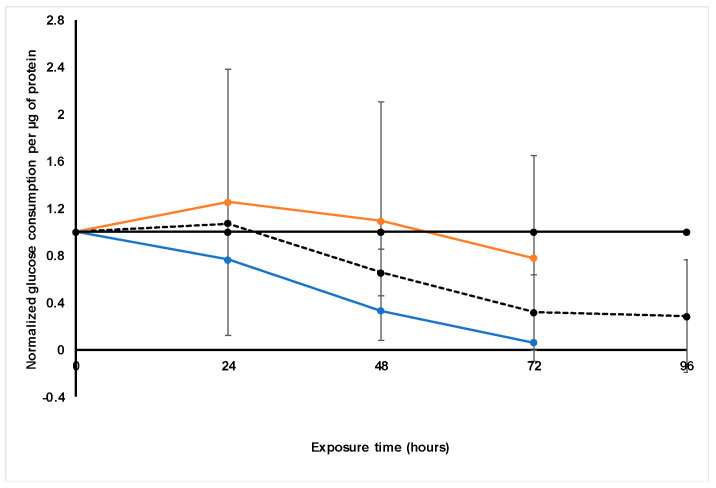
Normalized glucose consumption per microgram protein following exposure of the sodium alginate encapsulated LS180 mini-tumor model to *Sutherlandia frutescens* aqueous extract and paclitaxel. The solid black line represents the untreated control, the black dashed line represents paclitaxel [IC_50_], the orange line represents *S. frutescens* [IC_50_] and the blue line *S. frutescens* [IC_50_]/2. All data were normalized to the untreated control group (error bars = standard deviation; *n* = 3 for *S. frutescens* [IC_50_]/2; *n* = 6 for *S. frutescens* [IC_50_] and paclitaxel [IC_50_].

Through measuring the glucose clearance from the culture medium with a OneTouch^®^ Select^™^ blood glucose monitoring system, one can infer the approximate glucose consumption of cultured cells (mmol·L^−1^). This provides an estimate of metabolic activity of the cells. The data were again normalized to the glucose consumption per µg protein of the untreated control group, as displayed in Figure 5.

There was a clear and significant decrease in the levels of glucose consumed from 48 h onwards, following treatment with paclitaxel, relative to the untreated control group. From 72 h, very little glucose appeared to be consumed by the paclitaxel-treated LS180 spheroids [22].

*S. frutescens* appeared to increase the glucose content measured in the culture medium markedly, with a glucose content of 10.9 mmol·L^−1^ (±0.1) for the *S. frutescens* [IC_50_]/2 group and 16.8 mmol·L^−1^ (±0.1) for the *S. frutescens* [IC_50_] group, relative to the 6.8 mmol·L^−1^ (±0.1) glucose content of normal unspent medium.

Relative to the untreated control, however, the normalized glucose consumption per protein content decreased following treatment with the *S. frutescens* concentrations. This decrease in glucose consumption supported the conclusion that cell viability was decreased following treatment with *S. frutescens*. Glucose consumption could only be measured until 72 h in treatment with *S. frutescens*, as there are some technical problems with measuring glucose content in the presence of *S. frutescens* beyond 72 h. The reason for this is unclear and should be further investigated, but has been observed in different experiments and different cell lines.

### 2.4. Xysmalobium undulatum Anticancer Activity Screening in the LS180 Sodium Alginate Encapsulated Spheroid Model

The activity of *X. undulatum* against colorectal cancer was investigated by evaluating its effects on 12-day-old LS180 spheroids. The spheroids were treated for 96 h with [IC_50_] and 2[IC_50_] *X. undulatum* extract, which was replaced every 24 h. Soluble protein content, intracellular ATP content, extracellular AK release, and glucose consumption were measured as indicators of cell growth and viability relative to LS180 spheroids treated with [IC_50_] paclitaxel, the results of which were previously published in Smit et al. [22].

#### 2.4.1. Soluble Protein Content

With the Bradford assay, the soluble protein content of the LS180 spheroids was evaluated before and after treatment with *X. undulatum*. This indicated relative growth of the spheroids and enabled adjustment of the daily dose according to protein content.

**Figure 6:**
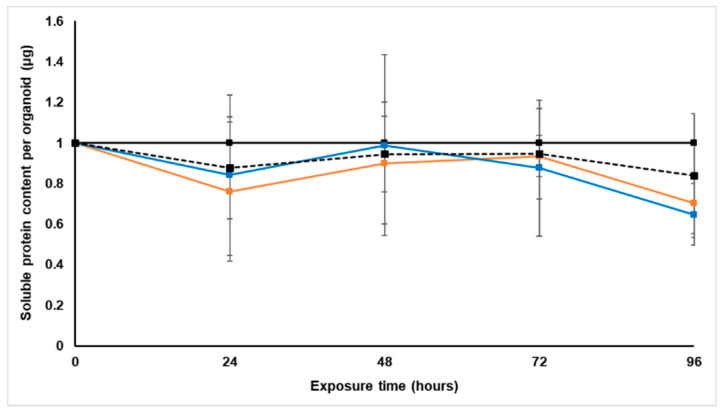
Normalized soluble protein content per spheroid (µg) of the sodium alginate encapsulated LS180 mini-tumor model, following 96 h exposure to *Xysmalobium undulatum* aqueous extract or paclitaxel. The solid black line represents the untreated control, the black dashed line represents paclitaxel [IC_50_], the orange line represents *X. undulatum* [IC_50_] and the blue line *X. undulatum* 2[IC_50_]. All data were normalized to the untreated control group (error bars = standard deviation, *n* = 6).

As illustrated in Figure 6, both *X. undulatum* treatment groups followed very similar trends to the paclitaxel-treated group [22]. All groups had slightly decreased soluble protein content relative to the untreated control group for the duration of the treatment.

The total soluble protein content per bioreactor was used as for the *S. frutescens* treatment, to calculate the daily dose for each treatment group based on the number of spheroids left and growth over time. The *X. undulatum* dosages per protein were established as 0.8 × 10^−2^ mg·mL^−1^·µg^−1^ protein for [IC_50_] *X. undulatum* and 1.50 × 10^−2^ mg·mL^−1^·µg^−1^ protein for the 2[IC_50_] *X. undulatum* group.

#### 2.4.2. Intracellular Adenosine Triphosphate Content

**Figure 7:**
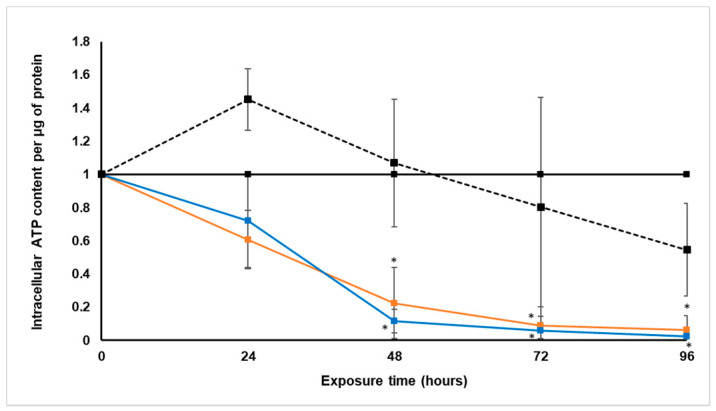
Normalized intracellular adenosine triphosphate content per soluble protein (µM·µg^−1^) following exposure of the sodium alginate encapsulated LS180 mini-tumor model to *Xysmalobium undulatum* aqueous extract and paclitaxel. The solid black line represents the untreated control, the black dashed line represents paclitaxel [IC_50_], the orange line represents *X. undulatum* [IC_50_] and the blue line *X. undulatum* 2[IC_50_]. All data were normalized to the untreated control group (error bars = standard deviation; *n* = 6; * = statistically significant, *p* < 0.05 (one-way ANOVA followed by the Dunnett post-hoc test).

During the treatment with *X. undulatum*, the intracellular ATP content was measured every 24 h with the CellTiter-Glo^®^ luminescence assay, as an indicator of cell viability. The ATP content was expressed relative to the soluble protein content and illustrated in Figure 7.

A steady decrease in the intracellular ATP content could be observed for both *X. undulatum* treatment groups, although the decrease was only significant after 48 h exposure. After 96 h, there was almost no ATP detectable. There was, however, very little difference in the ATP levels of the two *X. undulatum* treatment groups. Relative to the paclitaxel-treated group, the significant decrease in ATP levels suggests a much faster and more dramatic reduction in cell viability of the LS180 cells.

#### 2.4.3. Extracellular Adenylate Kinase Content

**Figure 8:**
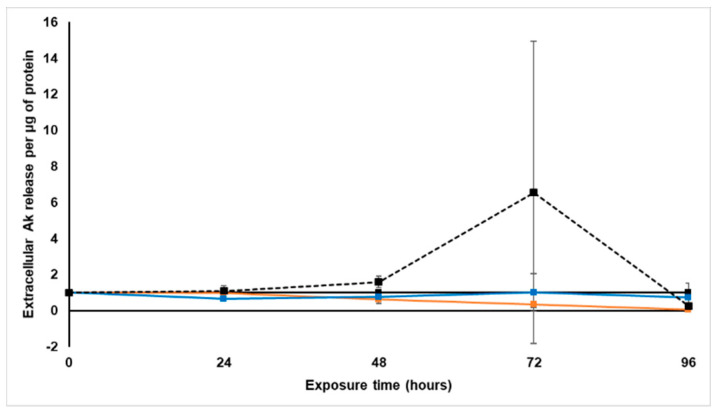
Normalized extracellular adenylate kinase release per microgram protein following exposure of the sodium alginate encapsulated LS180 mini-tumor model to *Xysmalobium undulatum* aqueous extract and paclitaxel. The solid black line represents the untreated control, the black dashed line represents paclitaxel [IC_50_], the orange line represents *X. undulatum* [IC_50_] and the blue line *X. undulatum* 2[IC_50_]. All data were normalized to the untreated control group (error bars = standard deviation; *n* = 6.

Following treatment with *X. undulatum* 2[IC_50_] and *X. undulatum* [IC_50_], the extracellular AK release per protein content was determined every 24 h with the ToxiLight^®^ luminescence BioAssay. These AK values were normalized and expressed relative to the untreated control, per µg protein (Figure 8).

The AK release for both *X. undulatum* treatment groups showed a slight decrease relative to the untreated control during the 96 h treatment. These changes were, however, not significant. When considering the soluble protein content and decrease in intracellular ATP observed, the results suggest that AK may have been released at time points not evaluated in this study.

#### 2.4.4. Glucose Consumption

**Figure 9:**
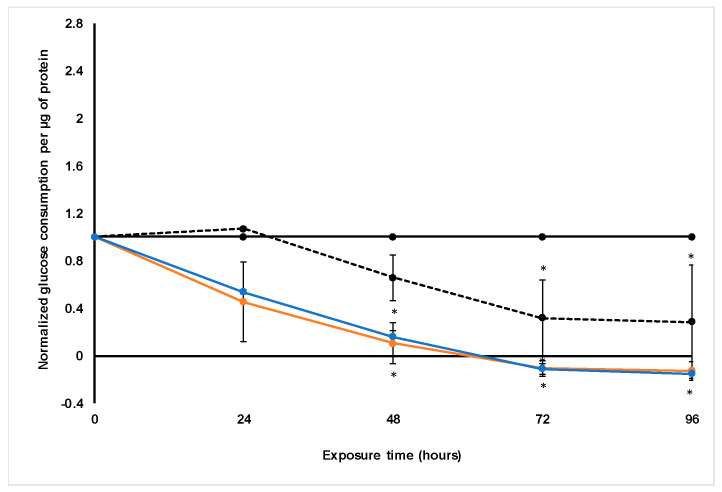
Normalized glucose consumption per microgram protein following exposure of the sodium alginate encapsulated LS180 mini-tumor model to *Xysmalobium undulatum* aqueous extract and paclitaxel. The solid black line represents the untreated control, the black dashed line represents paclitaxel [IC_50_], the orange line represents *X. undulatum* [IC_50_] and the blue line *X. undulatum* 2[IC_50_]. All data were normalized to the untreated control group (error bars = standard deviation; *n* = 6; * = statistically significant, *p* < 0.05 (one-way ANOVA followed by the Dunnett post-hoc test).

Glucose consumption per µg protein was estimated by measuring the clearance of glucose from the culture medium with a OneTouch^®^ Select^™^ blood glucose monitoring system. The glucose consumption was then normalized relative to the untreated control group (Figure 9).

*X. undulatum* appeared to also increase the glucose content measured in the culture medium slightly, with a glucose content of 7.2 mmol·L^−1^ (±0.1) for the *X. undulatum* [IC_50_] group and 7.8 mmol·L^−1^ (±0.1) for the *X. undulatum* 2[IC_50_] group.

Glucose consumption decreased steadily following treatment with both concentrations of *X. undulatum*, and this decrease was significant relative to the untreated control from 48 h exposure onwards. The trend was very similar to that of the paclitaxel-treated group [22], although treatment with *X. undulatum* decreased the glucose consumption faster and much more.

When taking into account the decreased soluble protein content and the intracellular ATP content, this decrease in glucose consumption corresponded well with the suggested cell viability and growth reduction due to treatment with *X. undulatum.*

### 2.5. Relative Cytochrome P450 Gene Expression

The CYP450 superfamily is the largest and most important phase I enzymes, and of these, the CYP3A, CYP2D and CYP2C families are responsible for respectively 50%, 25% and 20% of the biotransformation of all drugs [25]. Since various treatments can influence the expression of these enzymes, which in turn can affect the bioavailability of these treatments, it is an important parameter to evaluate when studying new potential treatments.

As shown in Smit et al. [22] and Li et al. [26], paclitaxel is known to upregulate the gene expression of CYP3A4 significantly (as much as 16-fold), and CYP2D6 to some extent as well. This, in turn, may lead to metabolic inactivation of the treatment [27]. These effects were found to be dose-dependent.

In this study, no expression of either CYP3A4 or CYP2D6 could be detected following treatment with either *S. frutescens* [IC_50_]/2 or the *S. frutescens* [IC_50_], even though the presence of the housekeeping genes glyceraldehyde 3-phosphate dehydrogenase (*GADPH*) and TATA-box binding protein (*TBP*) was indicated and present.

Similarly, following treatment with *X. undulatum* for 96 h, the qRT-PCR assay was performed and, although the housekeeping genes GADPH and TBP was indicated and present, expression of the CYP3A4 and CYP2D6 genes were not detected.

Expression of the CYP3A4 and CYP2D6 genes were confirmed in the untreated control samples.

## 3. Discussion

In this study, sodium alginate encapsulated LS180 spheroids were treated for 96 h with 0.6 × 10^−2^ µg·mL^−1^·µg^−1^ protein for paclitaxel [IC_50_], 20.1 × 10^−2^ mg·mL^−1^·µg^−1^ protein for *S. frutescens* [IC_50_]/2, 40.3 × 10^−2^ mg·mL^−1^·µg^−1^ protein for *S. frutescens* [IC_50_], 0.8 × 10^−2^ mg·mL^−1^·µg^−1^ protein for *X. undulatum* [IC_50_] and 1.5 × 10^−2^ mg·mL^−1^·µg^−1^ protein for *X. undulatum* 2[IC_50_], relative to an untreated control.

Treatment with *S. frutescens* [IC_50_]/2 decreased the soluble protein content relative to the untreated control, and the ATP content per µg soluble protein decreased below detectable limits. Except for a slight increase between 24 h and 72 h, the same decrease was observed for AK release per µg soluble protein. *S. frutescens* [IC_50_]/2 treatment also reduced glucose consumption to far less than that of the untreated group. These results were also found for the *S. frutescens* [IC_50_] treated group.

The observed decrease in cell growth and viability following treatment with *S. frutescens* aqueous extract suggests that this extract has cytotoxic effects on LS180 cells, and could have cancer treatment potential against colorectal cancer. The almost undetectable levels of ATP suggest cell death, since ATP levels fall as a cell dies [28]. ATP depletion probably occurred, and when combined with the lack of AK release, the presence of necrosis is very likely [29]. Although a previous study in Caco-2 cells cultured in 2D demonstrated induction of apoptosis following treatment for 48 h, but the study used an ethanolic extract [30]. Cytotoxicity of *S. frutescence* was also confirmed in DLD-1 colon cancer cells cultured in 2D, with IC_50_ values between 150 and 180 µg·mL^−1^ [31]. Furthermore, unlike paclitaxel, treatment with *S. frutescens* does not appear to induce gene expression of the CYP3A4 and CYP2D6 enzymes but rather maybe inhibits expression. Similar to a previous study in breast cancer cells [32], the results of this study suggest that the anticancer activity of the *S. frutescens* crude aqueous extract, specifically in colon cancer, may even surpass that of the model chemotherapeutic drug, paclitaxel. However, further studies are needed to understand the full extent of this activity, and the minimum concentration needed for activity.

*X. undulatum* [IC_50_] treatment for 96 h decreased soluble protein content relative to the untreated control, as well as the ATP content per µg soluble protein. Although the AK release per µg soluble protein was similar to that of the untreated group for the first 48 h of exposure, it then decreased and was nearly undetectable after 96 h. The glucose consumption also rapidly decreased after exposure, and this group consumed much less glucose relative to the untreated control. Treatment with *X. undulatum* 2[IC_50_] also resulted in decreased soluble protein content, ATP content per µg soluble protein, AK release per µg soluble protein and glucose consumption. Although a slightly higher AK release was observed for this treatment group.

*X. undulatum* crude aqueous extract could, therefore, also have anticancer potential, when considering the observed decrease in cell growth and viability. Calitz et al. [14] also previously suggested crude aqueous extracts of *X. undulatum* to have anti-proliferating effects, which was confirmed in the current study, although it must be confirmed if the effects are specific to cancer cells. The activity observed also surpassed that of the chemotherapeutic drug, paclitaxel when comparing the effects of the IC_50_ concentrations as determined in traditional culture, but the activity seemed to be less pronounced than that of *S. frutescens*.

Although both plant extracts show great promise, further comparative studies are needed to identify potential single active compounds from the plant extracts, which will better enable direct activity comparison with current treatment and will increase the potential for a marketable drug.

Lastly, the sodium alginate encapsulated LS180 mini-tumor model was successfully implemented to evaluate the anticancer potential of plant extracts against colorectal cancer. The potential use of African phytomedicines to address the burden of cancer on the continent requires thorough study in suitable models, and this study indicated that this model could be used for such evaluations.

## 4. Materials and Methods

### 4.1. Preparation and Characterization of the Plant Material Aqueous Extracts

*X. undulatum* (XU174) and *S. frutescens* (SFFW) plant material (dried and milled) were purchased from Afrinatural holdings (Prestige Laboratory Supplies CC, KwaZulu-Natal, South Africa). Crude aqueous extracts of *S. frutescens* and *X. undulatum* were previously prepared and characterized [15]. Briefly, both extracts were prepared at a 1:10 ratio in water (plant: liquid) followed by 45 min sonication at 45 °C. The plant material suspension was centrifuged at 5000× *g* for 10 min, and the supernatant collected. The process was repeated with the subsequent pellet, and the two supernatants combined. The supernatant was once again centrifuged at 1218× *g* for 5 min, before filtration through Whatman^™^ filtration paper with a pore diameter of 125 mm (Whatman^™^, Sigma-Aldrich, Johannesburg, South Africa). The collected filtrate was frozen at −80 °C before lyophilization in a Virtis freeze dryer (SP Scientific, Gardiner, NY, USA). The lyophilized material was powdered with a mortar and pestle.

The plant extracts were chemically characterized with ultra-performance liquid chromatography-mass spectrometry (UPLC-MS) analyses to enable the identification, comparison, and detection of the main active phytochemicals. Briefly, 2 mg of freeze-dried powder of *S. frutescens* and *X. undulatum,* respectively, was added to 2 mL of methanol; followed by 10 min sonication and filtration using a 0.2 μm syringe filter. Analysis was performed on a Waters Acquity UPLC system, with a photodiode array (PDA) detector coupled to a Xevo G2QToF mass spectrometer (Waters, Milford, MA, USA) and an Acquity UPLC BEH C18 column (150 mm × 2.1 mm i.d., 1.7 μm particle size, Waters), maintained at 40 °C as previously described [14,15].

The *S. frutescens* and *X. undulatum* extracts were prepared daily in pre-heated culture medium. The pH of the extracts was adjusted to the physiological range of normal pH (±7). The stock solutions were vortexed and sterilized using a 0.22 µm syringe filter. *S. frutescens* dilutions were prepared in culture medium in the range of 1 to 8 mg·mL^−1^, and *X. undulatum* from 0.05 to 0.5 mg·mL^−1^.

### 4.2. LS180 Cell Culturing

The LS180 cell line (cat. no. #CL-187^™^) was purchased from the American Tissue Culture Collection (ATCC, Manassas, VA, USA). Cells were cultured in low-glucose Dulbecco’s Modified Eagle’s medium (DMEM) (Gibco; Thermo Fisher Scientific, Johannesburg, South Africa), supplemented with 10% foetal bovine serum (FBS) (Gibco; Thermo Fisher Scientific, Johannesburg, South Africa), 2 mM L-glutamine (200 mM; Lonza; Whitehead Scientific (Pty) Ltd., Cape Town, South Africa), 1% non-essential amino acids (NEAA) (100 X; Sigma-Aldrich, Johannesburg, South Africa) and 1% penicillin/streptomycin (10,000 penicillin U·mL^−1^/10,000 streptomycin U·mL^−1^; Lonza, Whitehead Scientific, Cape Town, South Africa). Cells were cultured under standard conditions of 37 °C, 5% CO_2_ and 95% humidified air, and culture medium was replaced every second day. Cells were sub-cultured by means of scraping in culture medium.

### 4.3. The 3-(4,5-Dimethylthiazol-2-yl)-2,5-diphenyltetrazolium Bromide Cytotoxicity Assay

Prior to the MTT assay, LS180 cells were trypsinized with trypsin-ethylenediaminetetraacetic acid (EDTA) (Lonza; Whitehead Scientific (Pty) Ltd., Cape Town, South Africa), and seeded in clear 96-well plates (Corning Inc., Ascendis Medical, Johannesburg, South Africa) at 8000 cells per well, and left for 24 h to attach. Culture medium was removed 24 h post-seeding, and cells were treated with the various compounds at various concentrations in 200 μL of culture medium (0 h). Treatment-containing culture medium was replaced every 24 h, and the MTT assay performed after 96 h exposure as previously described [15,22].

Untreated wells (to indicate 100% cell viability), dimethyl sulfoxide (DMSO; Sigma-Aldrich Johannesburg, South Africa) background control wells and dead cell control wells (cells treated with 0.2% Triton X-100 to represent 97–100% cell viability inhibition) received fresh supplemented medium during medium exchanges. To ensure the extract caused no interference with the assay, a highest plant concentration control (8 mg·mL^−1^ for *S. frutescens* and 0.5 mg·mL^−1^ for *X. undulatum*) was also included.

At 96 h, the medium was removed from all the wells and the cells were washed twice with 100 μL phosphate-buffered saline (PBS) (1 X; HyClone, Separations, Johannesburg, South Africa). All wells (excluding the DMSO background control) received 180 µL non-additive medium, and 20 µL of a 5 mg·mL^−1^ thiazolyl blue tetrazolium bromide powder (Sigma-Aldrich, Johannesburg, South Africa) stock solution [33]. The 96-well plate was covered and placed on a compact rocker for 5 min, followed by further incubation for 4 h at 37 °C. The medium was carefully removed (except that of the highest plant concentration wells) and replaced with 200 µL DMSO, followed by incubation on a compact rocker for 1 h. The absorbance was measured at 560 nm, with a reference wavelength of 630 nm [34] using a SpectraMax^®^ plate reader (Paradigm^®^ Multi-Mode Detection Platform; Molecular Devices^®^; Separations, Gauteng, South Africa).

The MTT assays were performed in six-fold, and SPSS statistical analysis software (IBM SPSS Statistics for Windows, Version 25.0. Armonk, NY, USA: IBM Corp.), in conjunction with the Probit Analysis Method, were used to calculate IC_50_ values and 95% confidence limit ranges for *S. frutescens* and *X. undulatum*, using the data from the MTT analyses.

### 4.4. Culturing of the LS180 Sodium Alginate Encapsulated Spheroid Model

The method was described in detail in Smit et al. [22]. In brief, a 2.5% *w/v* sodium alginate (Sigma-Aldrich) solution was prepared in PBS, and the 50 mM CaCl_2_ and 150 mM NaCl cross-linker solution was prepared in distilled water.

Active clinostat bioreactors were purchased from Celvivo^®^ ApS (Odense, Denmark), and were prepared by filling the chambers and placing the bioreactors onto a drive-unit (BAM v4.6; CelVivo^®^ ApS, Odense, Denmark) in an incubator to rotate and equilibrate overnight at 37 °C and 5% CO_2_. LS180 cells were only trypsinized once before seeding, and the cell suspension was diluted to 2000 cells per µL. The cell suspension was centrifuged at 140× *g* for 5 min, and the cell pellet gently re-suspended in the prepared sodium alginate solution at 37 °C. The imbedded LS180 cells were pipetted as 1 µL droplets onto square Perspex blocks covered with hydrophobic paraffin film, and 0.5 µL of the cross-linker solution was added to each droplet. The petri dish was covered and incubated at room temperature for 5 min. Spheroids were transferred to the bioreactors in culture medium and the speed of each bioreactor was initially set at 16 rotations per minute (rpm). The culture medium was exchanged after three days of culturing during the first week, followed by medium exchange every second day thereafter. Rotation speed was adjusted daily to keep the spheroids suspended.

### 4.5. Anticancer Activity Screening in the LS180 Sodium Alginate Encapsulated Spheroid Model

The potential anticancer properties of *S. frutescens* and *X. undulatum* were investigated in the LS180 sodium alginate encapsulated spheroid model, relative to an untreated control and a standard chemotherapeutic drug, paclitaxel (Sigma-Aldrich) [22]. The treatments consisted of two separate experiments (except for [IC_50_]/2 *S. frutescens*) with three replicates per each biological treatment in each experiment. The treatment commenced when the spheroids were 12 days of age, and sampling took place following 0 h, 24 h, 48 h, 72 h and 96 h of exposure to the treatments. Experimental groups consisted of *S. frutescens* [IC_50_]/2 (half the concentration of *S. frutescens* per µg protein, based on the IC_50_ values obtained in the 2D cultures following the MTT assay), *S. frutescens* [IC_50_], *X. undulatum* [IC_50_] and *X. undulatum* 2[IC_50_] (two times the concentration of *X. undulatum* per µg protein, based on the IC_50_ values obtained in the 2D cultures following the MTT assay).

Culture medium containing the treatments were replaced every 24 h, after the measured soluble protein content was used to determine the dose per protein for the various treatment groups. The soluble protein content (µg) per spheroid was multiplied by the number of spheroids in each bioreactor per day to obtain a total protein mass (µg) for the bioreactor, and the doses were adapted accordingly. Since the spheroids are bioengineered with the sodium alginate base bio-design, size variation is minimal.

#### 4.5.1. Soluble Protein Content

One spheroid was sampled every 24 h from each treatment group and biological repeat, before replacing the treatment containing culture medium, to determine the soluble protein content using the Quick Start™ Bradford Protein assay (Bio-Rad, Lasec SA (Pty) Ltd., Midrand, South Africa). Each sampled spheroid was lysed by adding 450 µL water, and divided into three technical replicates in clear 96-well plates. Subsequently, 10 µL lysis buffer was added to each well, followed by 40 µL protein assay dye reagent. Following centrifugation at 1218× *g* for 2 min, the absorbance was measured at 595 nm with a Spectramax^®^ Paradigm plate reader. All samples were quantified relative to a bovine serum albumin (BSA) (Bio-Rad) standard.

#### 4.5.2. Intracellular Adenosine Triphosphate Content

One spheroid was sampled per treatment group every 24 h for each biological replicate, and placed in microcentrifuge tubes. All culture medium was removed, 300 µL PBS added and mixed vigorously. The lysate was divided into three technical replicates and plated in black Costar^®^ flat-bottom 96-well plates (Corning Inc., Ascendis Medical, Johannesburg, South Africa). CellTiter-Glo^®^ luminescent lysis buffer (100 µL) (Promega, Anatech Instruments (Pty) Ltd., Johannesburg, South Africa) was added to sample wells and mixed. The plate was covered and shaken for 40 min, followed by centrifugation at 1218× *g*. Luminescence was measured with a Spectramax^®^ Paradigm plate reader.

All samples were quantified relative to a known ATP standard (Sigma-Aldrich, Johannesburg, South Africa). The average intracellular ATP content was expressed as the normalized ATP content per soluble protein (µM·µg^−1^).

#### 4.5.3. Extracellular Adenylate Kinase Content

An amount of 200 µL of spent culture medium was sampled in microcentrifuge tubes from each treatment group (3 biological replicates of 200 µL each), at each time point prior to replacing the treatment containing culture medium. The medium was centrifuged at 140× *g* for 15 min, and 160 µL of the supernatant was then transferred to a new microcentrifuge tube. The transferred culture medium was again centrifuged at 15,000× *g* for 15 min, where after 140 µL of the supernatant was transferred to a new tube and flash-frozen in liquid nitrogen and stored at −150 °C until further use.

After samples were equilibrated to room temperature, 20 µL was plated per well in black 96-well plates with three technical replicates. Then 100 µL of AK detection reagent (ToxiLight^®^ BioAssay kit, Lonza, Whitehead Scientific (Pty) Ltd., Cape Town, South Africa) was added, and the covered plate placed on a compact rocker for 20 min. The plate was then centrifuged at 1218× *g* for 2 min, and the luminescence measured with a Spectramax^®^ Paradigm plate reader.

All samples were quantified relative to a known dead cell standard (Cyto-Tox Glo^®^ digitonin lysis buffer, Promega, Anatech Instruments (Pty) Ltd., Johannesburg, South Africa) to determine the number of dead cells per mL of culture medium.

#### 4.5.4. Glucose Consumption

The amount of glucose (mmol·L^−1^) in the spent medium was measured for each of the treatment groups, at each time point, following the second centrifugation step of the AK samples and the removal of 140 µL of the spent medium. The glucose content of each treatment containing culture medium sample was also measured before dosing to establish the unspent medium glucose content.

The glucose content was measured three times with a OneTouch^®^ Select^™^ blood glucose monitoring system and OneTouch^®^ Select^™^ test strips. Following calibration with the OneTouch Ultra^™^ control solution (Lifescan), 3 µL of the spent medium was loaded on the test strips by means of a pipette. The glucose concentrations were read and noted, and the average calculated.

By deducting the remaining glucose content from the glucose content in the unspent medium, an approximation of glucose consumption (mmol·L^−1^) for the various treatment groups per time point was determined.

### 4.6. Relative Cytochrome P450 Gene Expression

A qRT-PCR was used to investigate potential changes in the relative expression of *CYP3A4* and *CYP2D6*. Culture medium was removed from the sampled spheroids (3 to 5 per sample) and 200 µL RNAlater (Whitehead Scientific (Pty) Ltd., Cape Town, South Africa) solution was added to each sample. The samples were stored at −80 °C until further use.

Samples were thawed at room temperature and centrifuged at 400× *g* for 10 min. Total RNA was extracted using the PureLink^™^ RNA Mini Kit according to the manufacturer’s guidelines, followed by RNA quantification. Complementary deoxyribonucleic acid (cDNA) was synthesized with the High-Capacity cDNA reverse transcription kit (Applied Biosystems, Vilnius, Lithuania), using 2 µg of total RNA.

Real-time PCR was performed using TaqMan^™^ Fast advanced Master Mix (Thermo Fisher Scientific, Johannesburg, South Africa), according to the manufacturer’s guidelines, on the C1000Touch^™^ Thermal Cycler with 96-Well Fast Reaction Module (Bio-Rad, Singapore). Cycling conditions consisted of 2 min hold at 50 °C, 2 min hold at 95 °C, and then 40 cycles of 95 °C (1 s) and 60 °C (20 s) each. FAM-labelled TaqMan^™^ Gene Expression primers were used for the following genes: *CYP3A4* (Hs00604506_m1), *CYP2D6* (Hs00164385_m1), Glyceraldehyde 3-phosphate dehydrogenase (*GAPDH*) (Hs99999905_m1) and TATA-box binding protein (*TBP*) (Hs00427620_m1). *GAPDH* and *TBP* were used as housekeeping genes to normalize the data, and to guarantee the comparability of the calculated mRNA (messenger RNA) expression in all samples analyzed. All PCR analyses were performed in biological triplicates. Threshold cycle (Ct) values and data were further analyzed with Bio-Rad CFX Maestro Software v1.1 (Bio-Rad CFX Maestro. Ink). Relative gene expression was calculated using the 2-ΔΔCt method [35].

### 4.7. Statistical Data Analysis

Data collected were analyzed with Statistica^®^ (data analysis software system), version 13 (TIBCO Software Inc., 2018, http://statistica.io.) to determine statistical significance. One-way analysis of variance (ANOVA) followed with a Dunnett post-hoc test was conducted for comparison of multiple groups with a control group (untreated). Real-time PCR data were analyzed by means of one-way ANOVA with Bonferroni post-hoc test using Bio-Rad CFX Maestro 1.1 (4.1.2433.1219) software. Differences were considered statistically significant when *p* < 0.05.

## Figures and Tables

**Table 1:** Half maximal inhibitory concentration of cell metabolic activity (IC_50_) of the treatments, relative to an untreated control, in LS180 cells following 96 h exposure, as determined with the Probit analysis.

Treatment	Calculated 50% Inhibitory Concentration	95% Confidence Intervals
Paclitaxel ^1^	8.10 × 10^−5^ mg·mL^−1^ (94.595 nM)	6.90–9.20 × 10^−5^ mg·mL^−1^
*Sutherlandia frutescens*	2.63 mg·mL^−1^	2.46–2.77 mg·mL^−1^
*Xysmalobium undulatum*	9.80 × 10^−2^ mg·mL^−1^	9.00–10.50 × 10^−2^ mg·mL^−1^

^1^ Previously published in Smit et al. [22].

## Data Availability

The data presented in this study are available on request from the corresponding author.

## Supplementary Materials

The following are available online. Figure S1: Percentage cell viability inhibition (IC) relative to an untreated control following 96 h of exposure to different concentration of paclitaxel on the LS180 cell line, Figure S2: Percentage cell viability inhibition (IC) relative to an untreated control following 96 h of exposure to different concentration of *Sutherlandia frutescens* on the LS180 cell line, and Figure S3: Percentage cell viability inhibition (IC) relative to an untreated control following 96 h of exposure to different concentration of *Xysmalobium undulatum* on the LS180 cell line.

## Sample Availability

Samples of the *S. frutescens* and *X. undulatum* plant material are available from the corresponding author.
