# Supplementary material for: Anticancer Potential of Sutherlandia frutescens and Xysmalobium undulatum in LS180 Colorectal Cancer Mini-Tumors
Source: Molecules. 2021 Jan 25;26(3):605. doi: 10.3390/molecules26030605 (PMC7865898; doi:10.3390/molecules26030605)
Supplement: Supplementary file 1 [file molecules-26-00605-s001.pdf]

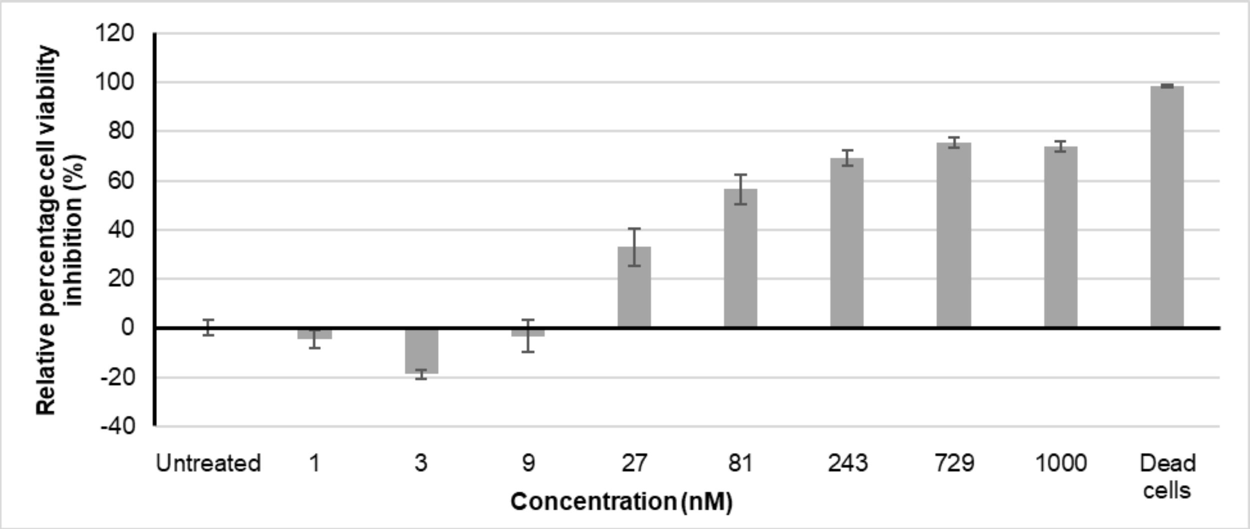

Figure S1. Percentage cell viability inhibition (IC) relative to an untreated control following 96 h of exposure to different concentration of paclitaxel on the LS180 cell line. (n = 6, error bars = standard deviation). The positive control consisted of cells treated with Triton X-100 (dead cells).

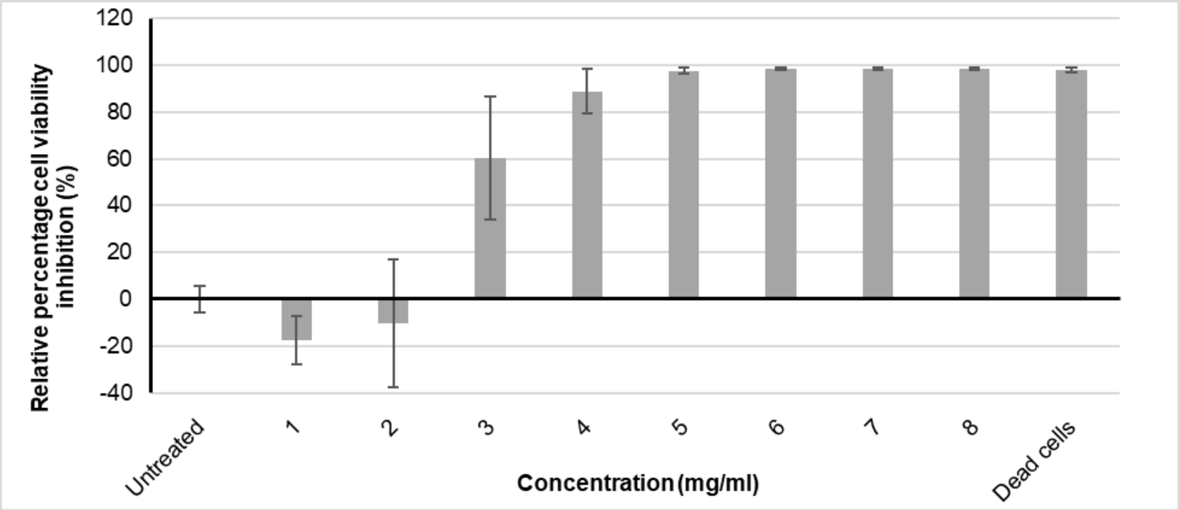

Figure S2. Percentage cell viability inhibition (IC) relative to an untreated control following 96 h of exposure to different concentration of *Sutherlandia frutescens* on the LS180 cell line. (n = 6, error bars = standard deviation). The positive control consisted of cells treated with Triton X-100 (dead cells).

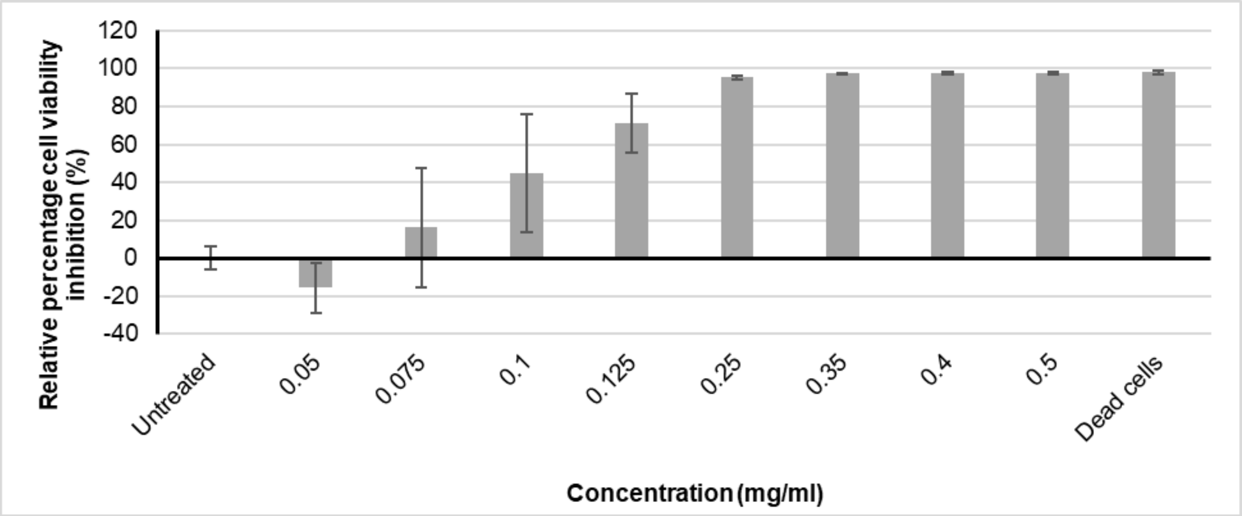

Figure S3. Percentage cell viability inhibition (IC) relative to an untreated control following 96 h of exposure to different concentration of *Xysmalobium undulatum* on the LS180 cell line. (n = 6, error bars = standard deviation). The positive control consisted of cells treated with Triton X-100 (dead cells).
